# The scent gland composition of the Mangshan pit viper, *Protobothrops mangshanensis*

**DOI:** 10.3762/bjoc.20.222

**Published:** 2024-10-18

**Authors:** Jonas Holste, Paul Weldon, Donald Boyer, Stefan Schulz

**Affiliations:** 1 Institute of Organic Chemistry, Technische Universität Braunschweig, 38106 Braunschweig, Germany; 2 Smithsonian Conservation Biology Institute, National Zoological Park, 1500 Remount Road, Front Royal, Virginia 22630, USA; 3 Bronx Zoo, 2300 Southern Boulevard Bronx, New York 10460, USA

**Keywords:** carboxylic acids, identification, mass spectrometry, pheromones, snakes

## Abstract

The Mangshan pit viper, *Protobothrops mangshanensis*, is a rare, highly endangered snake native to the mountainous regions of Hunan Province in China. Snakes possess abdominal scent glands, which have been chemically studied in several species. These glands can contain various lipids and peptides, but very often also complex mixtures of carboxylic acids. We report here the occurrence of novel methyl-branched unsaturated acids found in the secretions of six captive individuals living in a zoo. The structures of these compounds, 4.6-dimethylalk-5-enoates in a homologous series from C_11_–C_16_, were characterized by GC–MS and GC–IR analysis and various microderivatization reactions including hydrogenation and esterification leading to methyl and pyridylmethyl esters. In addition, dimethyloxazoline formation helped to localize the double bond. The synthesis of methyl 4,6-dimethyldodec-5-enoate allowed the correct assignment of structures and showed the (*E*)-configuration of the double bond for the major naturally occurring diastereomers. These acids occur in small amounts compared to the major glandular components, cholesterol, and 1-*O*-hexadecylglycerol, as well as other common long-chain alcohols and amides. Although a general defensive function has been proposed for snake abdominal scent glands, the specific chemistry and moderate amounts of acids reported here may suggest a function in chemical signaling for the Mangshan pit viper. In addition, proline-containing diketopiperazines were identified for the first time in snake scent glands, although an artificial formation from amino acids likely present in the secretion cannot be excluded.

## Introduction

Located in the tail base of all snakes is a pair of scent glands that open through ducts at the posterolateral margin of the vent. Snakes typically discharge pungent scent gland secretions (SGS) from the glands when disturbed, leading to the belief that these exudates deter predators like carnivores and others (see, e.g., [1–2]). Comparative analysis of SGS through GC–MS has revealed an assortment of lipids (reviewed in [2]), such as cholesterol [3–8], carboxylic acids [4–5,7–12], alcohols [8], 1-*O*-monoalkylglycerols [6,8], and others [13]. Nitrogen-containing compounds, including piperidone [10], amines [5,10], and amides [3,5,10] have been also observed in certain species.

The analyses of SGS in vipers (Viperidae) focused on New World pit vipers (Crotalinae). Early analyses of SGS lipids by thin-layer chromatography (TLC) [14], indicated that two pit vipers, the Timber Rattlesnake (*Crotalus horridus horridus*) and the Northern Copperhead (*Agkistrodon contortrix mokasen*), were devoid of neutral lipids. Subsequent TLC analysis of related snakes, such as the Eastern Diamondback Rattlesnake (*C. adamanteus*) and Florida Cottonmouth (*A. piscivorus conanti*), revealed the presence of various lipid classes including sterols, triacylglycerols, and common fatty acid methyl esters [15]. Mass spectrometric analysis of the lipid content of crotalines, mainly the Western Diamondback Rattlesnake (*C. atrox*), confirmed the presence of cholesterol [3,6,11,16], fatty acids [11], fatty amides [11], and 1-*O*-monoalkylglycerols of fatty acids from C_14_ to C_20_ [6]. A comparative analysis of the skin composition including the Old World vipers *Vipera ammodytes, V. berus*, *Montivipera bornmuelleri*, and *Daboia mauritanica* has been performed, revealing a multitude of different compounds including cholesterol and related steroids, as well as common fatty acids among other components. The contents of the SGSs were not investigated [13]. A comprehensive discussion of the SGS contents of snakes in general has been given by us before [2].

We report here the results of the analysis of the lipidic content of the SGS of an Old World crotaline, the Mangshan pit viper (*Protobothrops mangshanensis*), a venomous, arboreal snake endemic to a 105 km^2^ area of the subtropical forest around Mount Mang in the Nanling Mountain Range of the Hunan and Guangdong Provinces of southern China [17]. *P. mangshanensis* feeds mainly on small birds and rodents. It can attain total lengths of >2.1 m and weights of >5 kg, making it one of the largest Old World pit vipers. This protected species is endangered due to its restricted occurrence and increasing demand by illegal snake trade. We describe the characterization of the components of the SGS bouquet obtained from animals living in a zoo, including the identification of unique fatty acids of medium chain length not reported before from nature. Similar acids are not known from any other snake. In addition, small amounts of diketopiperazines were found for the first time in snake secretions.

## Results and Discussion

**Figure 1:**
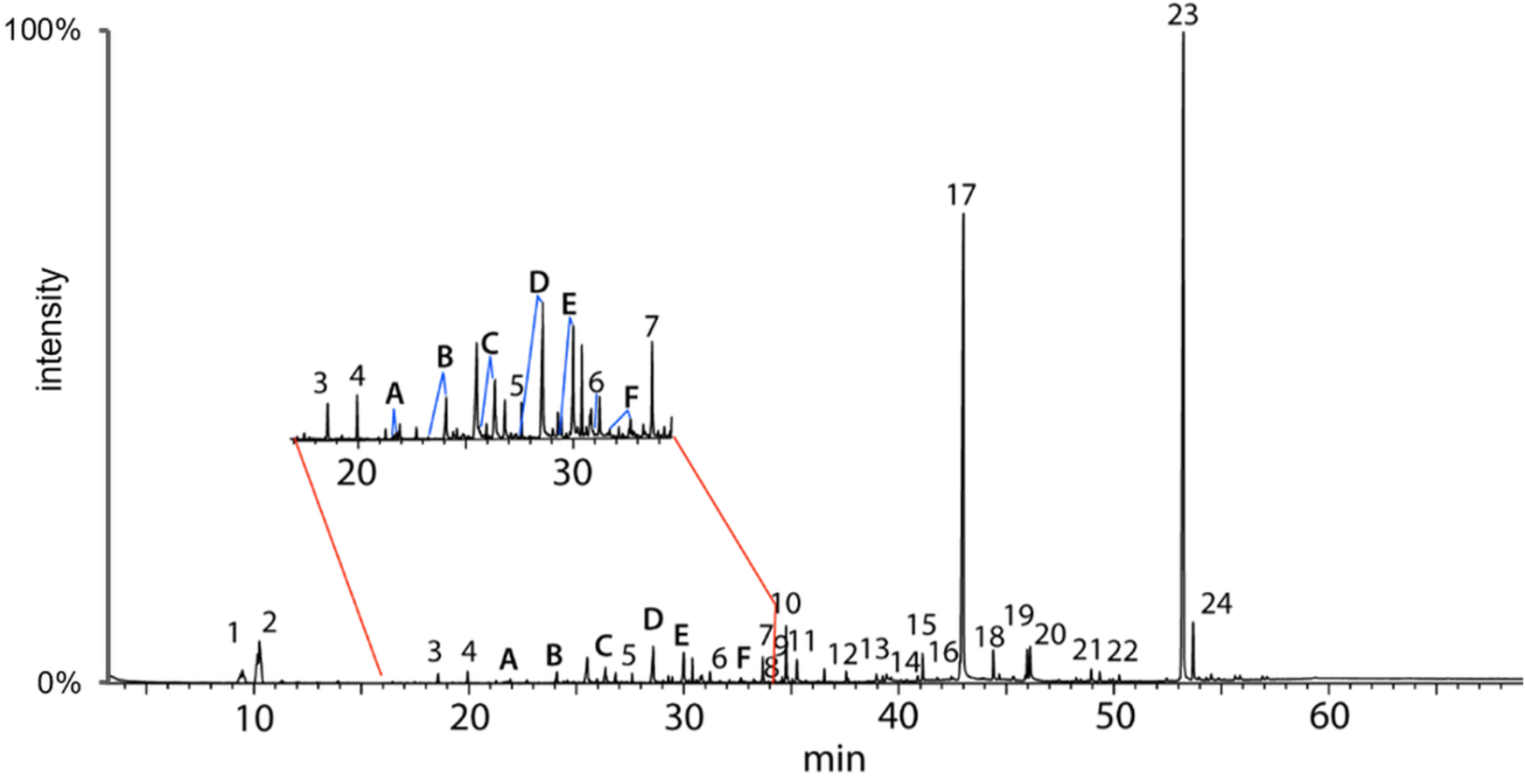
Total ion chromatogram of an extract of the scent gland of a Mangshan pit viper. Compounds **A**–**F** are the acids discussed in this article. Numbers refer to the compounds in Table 1.

GC–MS analysis of SGS extracts from six captive *Protobothrops mangshanensis* of both sexes revealed the presence of several classes of compounds that were consistently present in each sample. Besides cholesterol and the monoglyceride 1-*O*-hexadecylglycerol as major components, carboxylic acids, alcohols, glycerol ethers, amides, and volatile compounds such as phenol, benzaldehyde, or indole were present (Figure 1 and Table 1). While some of these compounds were easily identified, a group of diketopiperazines (DKPs) and a series of unknown putative carboxylic acids (**A**–**F**) were observed with similar mass spectra. The amount of secretion available and the complex mixture did not allow for the isolation of enough material for NMR analysis. Therefore, for the structure elucidation of these unknown compounds, we used different analytical methods, including GC–MS, GC–IR, and GC–HRMS. Additional chemical derivatization of the extracts and final synthesis of the proposed structure candidates led to the structures of compounds **A**–**F**.

**Table 1:** Compounds identified in Mangshan pit viper SGS extracts. Numbers refer to Figure 1. *I*: linear gas chromatographic retention index. The compounds were detected in all six samples analyzed.

No.	*I*	Compound

1	953	benzaldehyde
2	978	phenol
3	1221	benzothiazole
4	1291	indole
5	1564	dodecanoic acid
6	1809, 1833	cyclo(valyl-proline)
7	1877	1-hexadecanol
8	1905, 1937	cyclo(isoleucyl-proline)
9	1924	cyclo(leucyl-proline)
10	1933	cyclo(prolyl-proline)
11	1961	hexadecanoic acid
12	2081	1-octadecanol
13	2161	octadecanoic acid
14	2224, 2238	cyclo(methionyl-proline)
15	2284	1-eicosanol
16	2324	cyclo(phenylalanyl-proline)
17	2399	1-*O*-hexadecylglycerol
18	2488	1-docosanol
19	2593	eicosanamide
20	2602	1-*O*-octadecylglycerol
21	2804	docosanamide
22	2833	squalene
23	3117	cholesterol
24	3151	desmosterol

### Unknown compounds **A**–**F**

**Figure 2:**
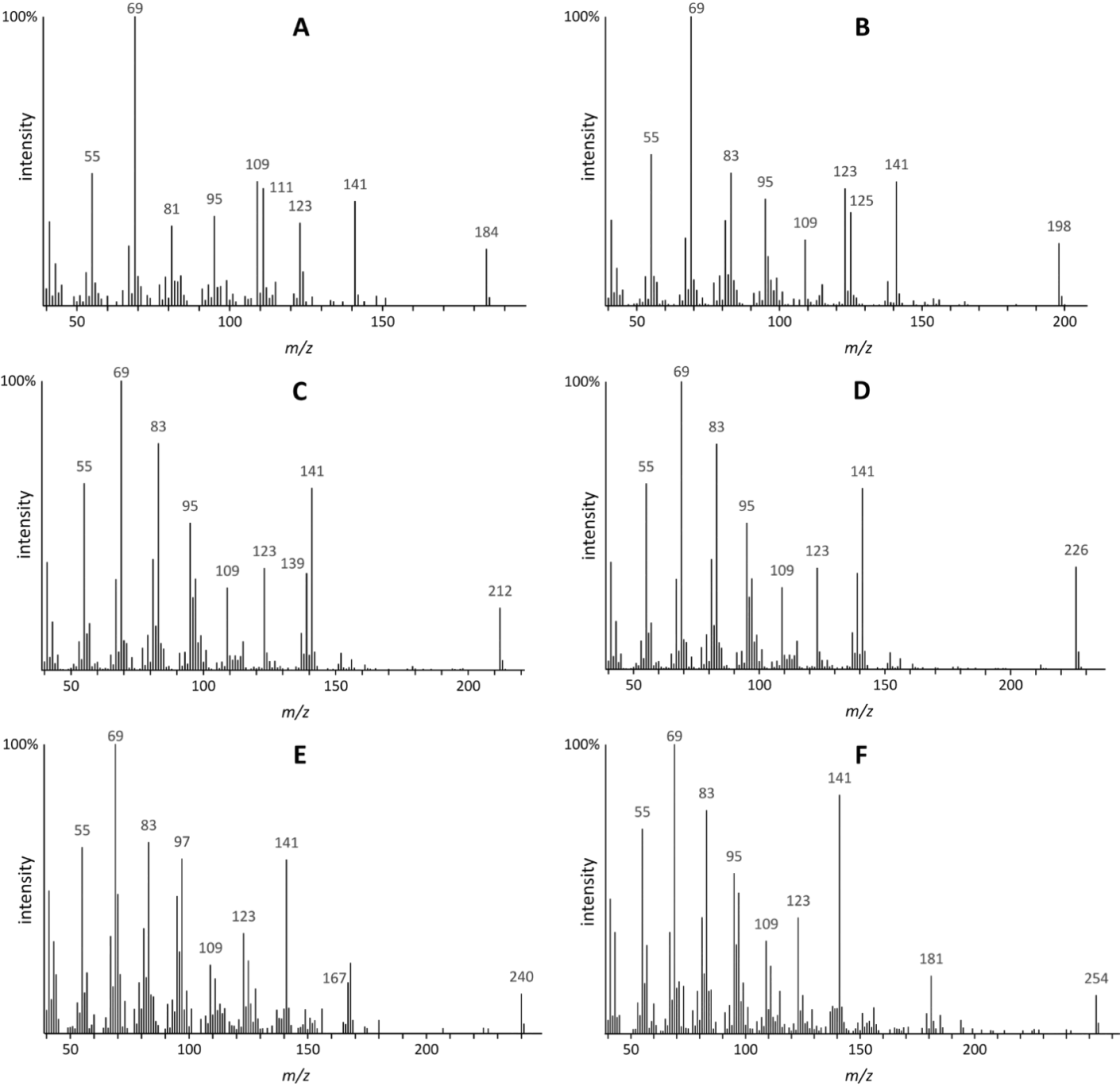
Mass spectra of compounds **A–F** show characteristic similarities with *m*/*z* 141 and ions of the series *m*/*z* 41, 55, 69, 83 and *m*/*z* 95, 109, 123. The molecular ion increases stepwise by one methylene unit.

Compounds **A**–**F** showed closely related mass spectra (Figure 2) and mostly occurred as pairs of diastereomers, labeled, e.g., **B** and **B’** here. The molecular ion and one of the fragment ions both increased by 14 Da, respectively, within the series. This suggests that **A**–**F** have the same core structure, but different chain lengths. Although the mass spectra showed small ions *m*/*z* 60 and 73 characteristic for aliphatic carboxylic acids, comparison with in-house and other spectral libraries revealed no close match with known compounds. High-resolution EI gas chromatography (HREI–GC–MS) of compound **D** suggested a molecular formula of C_14_H_26_O_2_ (*m*/*z* 226.18962 [M]^+^, calcd 226.19328), and two double-bond equivalents. The characteristic ion at *m*/*z* 141 showed a composition of C_8_H_13_O_2_ (*m*/*z* 141.0898, calcd. 141.0910).

Derivatization with *N-*methyl-*N*-(trimethylsilyl)trifluoroacetamide (MSTFA) or trimethylsulfonium hydroxide (TMSH) converted **A**–**F** into the corresponding trimethylsilyl or methyl esters (**Am**–**Fm**) (Supporting Information File 1, Figure S1), confirming the acid functional group in the natural compounds. In support of these data, GC/IR analysis of **Dm** (Supporting Information File 1, Figure S2) showed strong carbonyl bands at 1741 cm^−1^ accompanied by two intermediate bands at 1198 cm^−1^ and 1177 cm^−1^, characteristic of ester valence vibrations. The linear gas chromatographic retention index (*I*) of **Dm** was 1595, lower than, e.g., 1621 reported for methyl 2,11-dimethyldodecanoate [18], indicating a multi-branched carbon chain. The number and positions of methyl groups along the chain can be estimated using the empirical calculation system for *I* that we developed earlier [18–19]. While *I* for methyl dodecanoate is 1531, the small difference of 64 from the value of **Dm** indicates two methyl or even an ethyl substituent in addition to a double bond in **D**.

Therefore, the derivatized extract containing **Dm** was hydrogenated in the hope that the saturated compound would provide more insight into the structure. After hydrogenation, the spectra of methylated and hydrogenated **Dmh** showed *m*/*z* 87 as the base peak (Supporting Information File 1, Figure S3), which is strongly characteristic of a methyl branch at C-4 [20]. The position of the second methyl branch could not be determined due to the decreasing peak intensity with increasing fragment chain length.

To determine the position of the double bond position, we performed a microderivatization with dimethyl disulfide (DMDS) of a methylated extract. Unfortunately, we were unable to detect the derivatization products by GC–MS. Failure of DMDS derivatization on unsaturated isoprenoids has been reported [21]. It has been suggested that steric hindrance of trisubstituted double bonds results in little or no adduct formation [21].

Another method for elucidating double bond positions is the formation of 4,4-dimethyloxazoline (DMOX) derivatives [22–23]. DMOX-derivatized unsaturated fatty acids give characteristic spectra that often allow the location of the double bond position. The spectra of DMOX-derivatized compounds **Bd**–**Fd** showed a very high peak at *m*/*z* 113 and also characteristic ions at *m*/*z* 126, 167, and 208 (Figure S4 in Supporting Information File 1). We compared these spectra with those published by Christie et al*.* on various octadecanoate DMOX derivatives [23–24]. The peak pattern of **Bd**–**Fd** showed high similarity to that of the DMOX derivative of 5-enoates. Characteristic ions for derivatives with a C-5 double bond are a base peak at *m*/*z* 113 and two characteristic ions, *m*/*z* 153 resulting from formal cleavage of the double bond, and *m*/*z* 180, resulting from allylic cleavage of the C-7–C-8 bond (Supporting Information File 1, Figure S5) [23–24]. A similar pattern can be seen in the spectra of **Bd–Fd** but with partially shifted ions (Supporting Information File 1, Figure S4). Peak *m*/*z* 113 is present, but peak *m*/*z* 153 is shifted to *m*/*z* 167, while *m*/*z* 180 is shifted to *m*/*z* 208. The ion *m*/*z* 126 indicates a methylene group at C-3. These data suggest that an additional methyl group is located at C-4 or C-5, a second one is located at C-6 or C-7, and a double bond at C-5.

The positional ambiguities were resolved by the derivatization of a crude extract with pyridin-3-ylmethanol, yielding pyridylmethyl esters [8,25]. The spectrum of the pyridylmethyl ester derivative **Dp** confirmed that the first methyl group was at C-4, due to a missing peak at *m*/*z* 178 in the corresponding ion series (Supporting Information File 1, Figure S6). The next visible ion in this series, *m*/*z* 232 results from cleavage adjacent to the double bond, and the ion *m*/*z* 246 results from allylic cleavage. The 40 Da gap between *m*/*z* 192 and 232 indicates a methyl group either at either C-5 or C-6. Taken together, the data from the derivatizations led to the conclusion that one methyl group is located at C-4 and a second at C-6. This is consistent with regular fatty acid biosynthesis of mid-chain methyl substituents, which are usually separated by an odd number of carbons and located at even-numbered carbons due to their biosynthetic origin from methylmalonate units [26].

**Figure 3:**
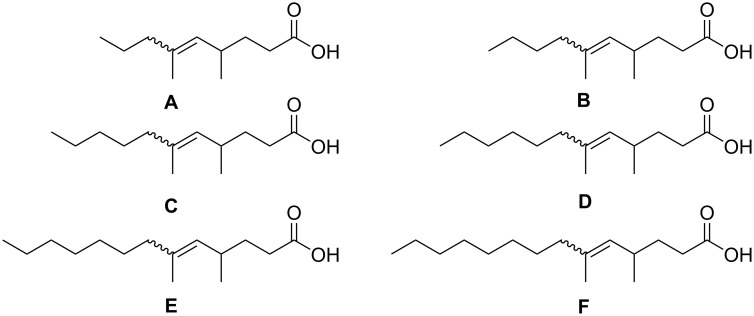
Structural proposals for compounds **A**–**F**.

From these analytical data, we proposed that compounds **A–F** were 4,6-dimethyl-5-enoic acids, ranging from C_9_ to C_14_ in chain length (Figure 3). Since **A–F** were obviously a homologous series of compounds, we synthesized compound **D** as its methyl ester **Dm** to compare the mass spectrum and *I* with those of the methyl esters of the natural acids.

**Scheme 1:**
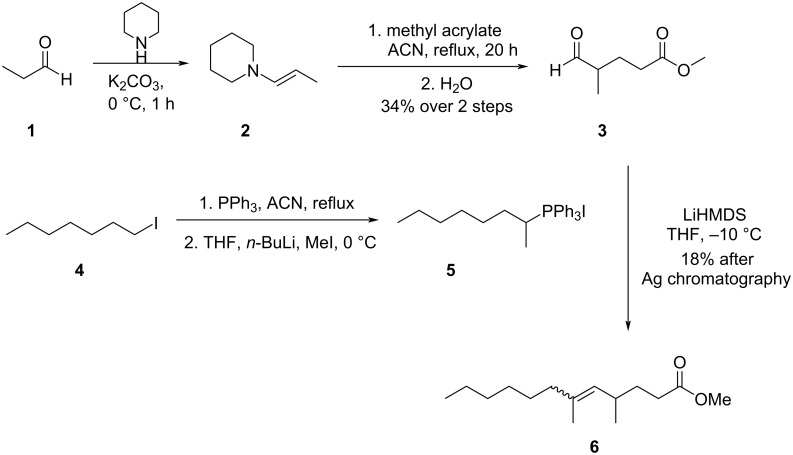
Synthesis of methyl 4,6-dimethyldodec-5-enoate (**6**). ACN: acetonitrile.

**Figure 4:**
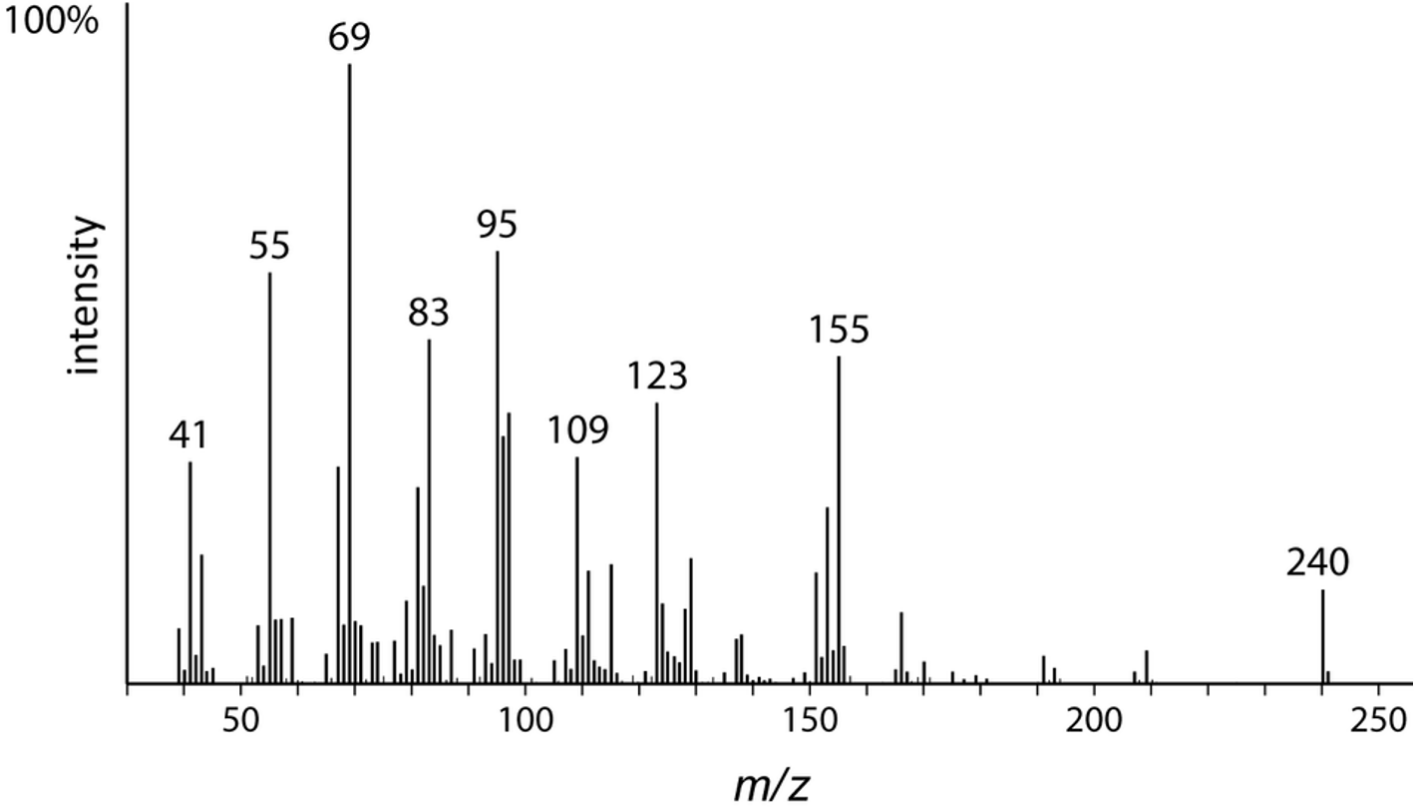
Mass spectrum of synthetic methyl (*E*)-4,6-dimethyldodec-5-enoate (*E*-**6**), identical with compound **D**.

Methyl 4,6-dimethyldodec-5-enoate (**6**) was synthesized as a mixture of *E*/*Z* isomers (Scheme 1) starting from the oxoester **3**, which is conveniently available from propenylpiperidine (**2**) [27] and methyl acrylate [28]. The Wittig reagent 1-methylheptyltriphenylphosphonium iodide (**5**) was prepared by methylation of the Wittig salt of heptyl iodide (**4**). A Wittig reaction with **3** afforded an *E/Z* mixture of methyl 4,6-dimethyldodec-5-enoate (**2**). The mass spectra of these compounds were identical to those of methyl ester **Dm** (Figure 4). The values of *I* were 1570 and 1595, in exact agreement with those of **Dm** and **Dm’**, confirmed by coinjection (Supporting Information File 1, Figure S7).

The two synthetic diastereomers were then subjected to silver nitrate column chromatography to separate the geometric isomers. This separation was only partially achieved (Supporting Information File 1, Figure S10), but one diastereomer was enriched and the sample was subjected to NOESY NMR experiments. Coupling was observed between H-5 (4.82 ppm) and C-6-methyl (1.66 ppm) (Supporting Information File 1, Figure S11) for the minor compound eluting earlier in GC, indicating that both hydrogen atoms are close together and that the double bond has a *Z*-configuration. The other diastereomer showed no coupling between H-5 (4.84 ppm) and C-6-methyl (1.56 ppm) and was assigned as the *E*-diastereomer. This assignment is further supported by the ^13^C NMR spectra. The major diastereomer shows C-7 at 39.7 ppm, a typical value for (*E*)-configured aliphatic chains with allylmethyl groups [29], while the (*Z*)-isomers show values around 30 ppm. The major component **Dm** of the methylated secretion with *I* 1595 was therefore assigned to have the *E* configuration. The *E*/*Z* ratio **Dm**/**Dm’** in all samples was about 20:1. In all cases the second eluting diastereomer of compounds **Am**–**Fm** was present in higher proportions, indicating a preferred *E*-configuration for all compounds except **Em**, where both diastereomers were present in approximately equal amounts.

We conclude that (*E*)-4,6-dimethyldodec-5-enoic acid is **D**, accompanied by **D’**, which is the (*Z*)-diastereomer. As shown, the spectra of natural products **A**–**C**, **E,** and **F** are very similar to **D**, except for the ions that depend on the backbone chain length. The same is true for the respective methyl esters; their mass spectrometric fragmentation is shown in Supporting Information File 1, Figure S12. The natural acids thus form a homologous series ranging from 4,6-dimethylnon-5-enoic acid (**A**) to 4,6-dimethyltetradec-5-enoic acid (**F**, Table 2). No differences between samples from males and females were detected.

**Table 2:** Acids in the secretion of the Mangshan pit viper and gas chromatographic retention indices *I* of compounds **A**–**E** and **Am**–**Em**.

Acid	Compound	*I* *_acid_*	Ester	*I*_methyl ester_

**A'**	(*Z*)-4,6-dimethylnon-5-enoic acid	1348	**Am'**	not found
**A**	(*E*)-4,6-dimethylnon-5-enoic acid	1360	**Am**	1316
**B'**	(*Z*)-4,6-dimethyldec-5-enoic acid	1430	**Bm'**	1387
**B**	(*E*)-4,6-dimethyldec-5-enoic acid	1450	**Bm**	1405
**C'**	(*Z*)-4,6-dimethylundec-5-enoic acid	1520	**Cm'**	1477
**C**	(*E*)-4,6-dimethylundec-5-enoic acid	1542	**Cm**	1499
**D'**	(*Z*)-4,6-dimethyldodec-5-enoic acid (*Z*-**6**)	1610	**Dm'**	1570
**D**	(*E*)-4,6-dimethyldodec-5-enoic acid (*E*-**6**)	1638	**Dm**	1595
**E'**	(*Z*)-4,6-dimethyltridec-5-enoic acid	1701	**Em'**	1661
**E**	(*E*)-4,6-dimethyltridec-5-enoic acid	1730	**Em**	1692
**F'**	(*Z*)-4,6-dimethyltetradec-5-enoic acid	1781	**Fm'**	not found
**F**	(*E*)-4,6-dimethyltetradec-5-enoic acid	1827	**Fm**	1790

As discussed in the introduction, fatty acids are common constituents of the SGS of many snakes and show great structural diversity. Their typical chain length is between 2–26 carbons and they have been found in pythonids, elapids, viperids, boas, and colubrids [3–5,7,9–11]. In some species, methyl-branched or unsaturated fatty acids have been detected [5,8,10], but to the best of our knowledge, 4,6-dimethyl fatty acids of medium chain length have not been reported from nature. Their formation requires a finely tuned biosynthetic machinery that deviates from the common fatty acid biosynthetic pathway operating, also in snakes, that we discussed earlier [8].

Fatty acids in SGS have been proposed to act as insecticides against ants or other harmful arthropods as they are thought to be secreted for defense [9,30]. However, the use of acids as defensive agents would likely not require structurally relatively highly tuned compounds, and compounds **A**–**F** are only produced in moderate amounts. The structural uniqueness, the moderate amounts found, and the relatively smaller size compared to common fatty acids, which allow for easier evaporation, make acids **A**–**F** suitable to be used in chemical communication of *P. mangshanensis*. However, actual proof of function can only be obtained by bioassays with synthetic material. Interestingly, there are a few structurally related compounds in the animal kingdom that serve as signals, such as the aldehydes *syn*-4,6-dimethylundecanal and 4,6-dimethyldodecanal and their corresponding alcohols, which act as male sex and trail-following pheromones in termites [31–32].

### Diketopiperazines

**Figure 5:**
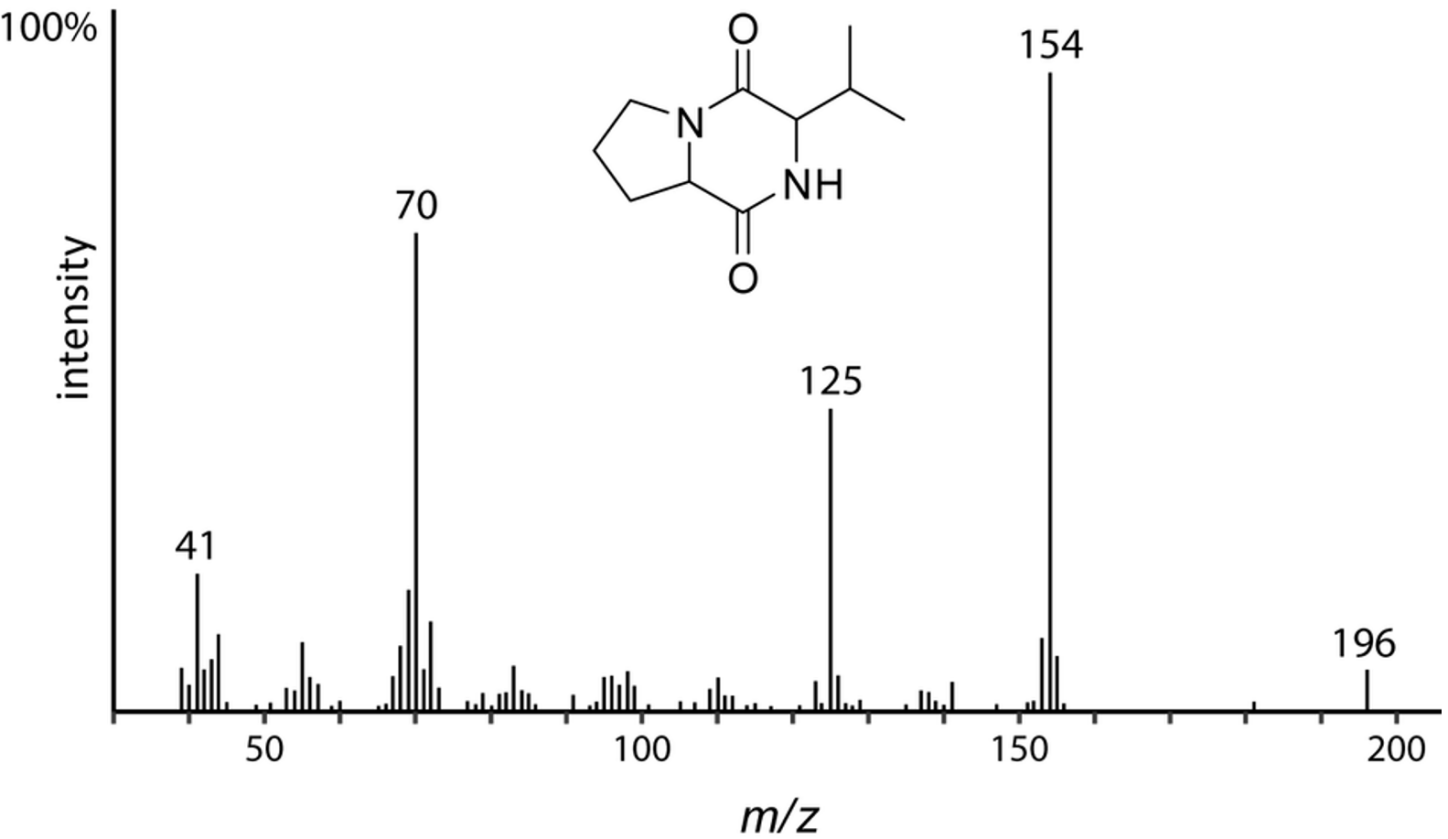
Mass spectrum of cyclo(valyl-proline).

The GC–MS analysis also revealed the presence of at least five diketopiperazines (DKPs) in the SGS, all containing proline. Fragments *m*/*z* 70 [C_4_H_8_N]^+^ and *m*/*z* 154 [C_7_H_10_O_2_N_2_]^+^, which are characteristic of diketopiperazines of proline, showed intense peaks. The mass spectrum of *cyclo*(valyl-proline) is shown in Figure 5.

In summary, six DKPs were identified by their characteristic mass spectra [33], cyclo(valyl-proline), cyclo(leucyl-proline), cyclo(isoleucyl-proline), cyclo(prolyl-proline), cyclo(phenylalanyl-proline), and cyclo(methionyl-proline) (Table 1). Most of the DKPs occurred as a mixture of diastereomers with two peaks in the chromatogram.

Although naturally occurring DKPs usually consist only of the ʟ,ʟ-diastereomer, here both *syn*- and *anti*-diastereomers were observed. This may be due to slow epimerization due to enolization within the gland or during sample preparation and storage, as the ᴅ,ʟ-diastereomers of proline DKPs are more stable than the ʟ,ʟ-diastereomers [34]. Unfortunately, the absolute configuration of these compounds could not be determined due to the small amounts available. Diketopiperazines can be formed either enzymatically or non-enzymatically, e.g., by heat dimerization of amino acids [35]. Both antibacterial and antifungal activities have been described for DKPs, but cyclo(ᴅ-phenylalanyl-ᴅ-proline) can also serve as a signal molecule regulating gene expression [35–38] and cyclo(prolyl-proline) is a pheromone of diatoms [39].

### Other compounds

Many of the other compounds detected in this study have also been reported in the secretions of other snakes and reptiles. The most abundant compound cholesterol is a ubiquitous lipid in reptile skin and glands [2]. 1-*O*-Hexadecylglycerol is the second most abundant SGS component. Such ether lipids have previously been reported from the crotaline *Crotalus atrox* [6] or the phyton *Loxocemus bicolor* [8]. These ethers are also found in the scent glands of other reptiles such as lizards [40] and probably act as detergents to increase the solubility of apolar lipids. Amides have been found in Dumeril's Boa and the western diamondback rattlesnake, among others [5,11]. The secretion has also been investigated by direct inlet atmospheric solids analysis probe-atmospheric mass spectrometry (ASAP-APCI-MS), which allows the detection of secondary metabolites up to a molecular mass of 1,500 Da, but no additional compounds were detected. A more detailed description of the use of this method in natural product research has recently been published by our team [41].

## Conclusion

4,6-Dimethylalk-5-enoic acids are important components of the scent gland secretion of *Protobothrops mangshanensis.* Since NMR analysis was not possible due to the low amounts present, the compounds were identified only with the help of several microderivatizations and finally confirmed structurally by synthesis. The structural uniqueness of the compounds, not found in other snake species, suggests a function, may it be in chemical communication, defense, or other traits. The other scent gland constituents are commonly found in other snakes except for the proline-derived diketopiperazines.

## Experimental

### General procedures

IR spectra were recorded on an Agilent Technologies 7890B gas chromatograph coupled to a Dani Instruments DiscovIR DD-FTIR solid phase interface. Chromatography was performed on an Agilent HP5MS capillary column (30 m × 0.25 mm, 0.25 µm film thickness) with helium as the carrier gas. The eluted compounds were applied to a ZnSe disk rotating at 4 mm/min at −40 °C. The resulting IR spectra were processed using GRAMS/AI 9.2 software (Thermo Fisher Scientific). High-resolution mass spectrometry data were obtained using a GC 6890 (Agilent Technologies) coupled to a JMS-T100GC mass spectrometer (GC-AccuTOF, JEOL, Japan) using the time-of-flight (70 eV) method. A ZB5 MS column (Phenomenex, 30 m, 0.25 mm i.d. 0.25 µm film thickness) and helium as carrier gas were used. NMR spectra were obtained using Bruker DPX300, AVIIIHD500, or AVII 600 instruments. Chemical shifts were measured against an internal standard, tetramethylsilane (TMS, δ = 0 ppm). GC–EIMS analyses were performed on either an HP6890 gas chromatograph coupled to an HP5973 mass spectrometer or an Agilent 7890B gas chromatograph coupled to an Agilent 5977A mass spectrometer. Agilent HP5-MS columns (Agilent Technologies, 30 m × 0.25 mm, 0.25 µm film thickness) were used as stationary phases, and helium was used as the carrier gas. EI-ionization was performed at 70 eV. The temperature program was as follows for both GC–MS and GC–IR: 50 °C (5 min isothermal), increasing at 5 °C/min to 320 °C, which was held isothermal for 5 min, or 50 °C (5 min isothermal), increasing at 20 °C/min to 320 °C, which was held isothermal for 5 min. Gas chromatographic linear retention indices were calculated based on a homologous series of *n*-alkanes (C_8_–C_30_) analyzed under identical conditions. All chemicals were purchased from Sigma-Aldrich, VWR, Acros Organics, Alfa Aesar, Carl-Roth, or Merck. Derivatizations and extractions were performed with solvents for GC (Merck, Suprasolv). Deuterated solvents for NMR were purchased from Deutero. Solvents for synthesis were distilled before use. All other chemicals were used without purification unless otherwise stated. Air- and H_2_O-sensitive reactions were performed under N_2_. ASAP-APCI-MS was performed on an Expression MS (Advion) mass spectrometer equipped with an APCI ionization source operated in positive mode. The capillary temperature was 250 °C, the capillary voltage was 180 V, the source voltage offset was 20 V, and the source voltage span was 30 V. The APCI source gas temperature was 350 °C and the APCI corona discharge was set to 5.0 µA. The scan range was 118 amu to 1522 amu. A direct probe, a small glass rod, was inserted into the secretion sample and then directly into the ASAP ionization chamber.

### Scent glands

Scent gland secretions of *P. mangshanensis* were collected from three adult males (1.3–1.7 m, X = 1.5 m) and three adult females (1.3–1.7 m, X = 1.5 m) living in the Bronx Zoo, New York, USA. All snakes were captive-born and fed regularly with mice. Snakes were restrained while manual pressure was applied to the base of the tail. The emerging stream of brown liquid, roughly 0.2 mL, was directed into glass vials to which 3 mL CH_2_Cl_2_ were added. The samples were kept frozen until analysis. The natural extract was filtered through a glass pipette plugged with Kimtech paper before GC–MS analysis and derivatization.

### Extract derivatization

**Silylation with MSTFA:** An extract (100 µL) was mixed with 50 µL of *N-*methyl-*N*-(trimethylsilyl)trifluoroacetamide (MSTFA) in a 2 mL GC vial. The solution was heated to 60 °C for 60 min and then excess MSTFA was removed with a stream of N_2_. The sample was taken up in CH_2_Cl_2_ and analyzed by GC–MS [42].

**Methylation with TMSH:** An extract (100 µL) in a 2 mL GC vial was mixed with 50 µL trimethylsulfonium hydroxide in methanol (TMSH, 0.25 M) and allowed to stand at room temperature for 1 h. The solvent was removed with a stream of N_2_ and the sample was taken up in CH_2_Cl_2_ and analyzed by GC–MS [43].

**Dimethyl disulfide derivatization:** Twenty µL of the sample in a 2 mL GC vial were diluted with pentane (20 µL) and mixed with freshly distilled dimethyl disulfide (DMDS, 50 µL) and a 0.24 M iodine solution in diethyl ether (5 µL). The mixture was allowed to stand sealed at 40 °C for 15 h. Subsequently, the mixture was diluted with pentane (200 µL) and washed with a saturated sodium thiosulfate solution. The organic phase was dried over sodium sulfate and concentrated under a stream of N_2_.

**Hydrogenation:** The solvent of the natural extract (100 µL) was removed with a stream of N_2_ and taken up in pentane (100 µL) and a catalytic amount of Pd/C was added. The reaction was then stirred for 1 h under a H_2_ atmosphere. The catalyst was filtered and rinsed with pentane. The extract was concentrated by slow evaporation at room temperature before analysis.

**Derivatization with 2-amino-2-methyl-1-propanol (DMOX derivatives):** 2-Amino-2-methyl-1-propanol (50 µL) was added to the natural extract (100 µL) in a 2 mL GC vial. The vial was purged with N_2_ and heated to 120 °C for 18 h. After cooling down to room temperature, water, and diethyl ether were added. The phases were separated and the aqueous phase was extracted three times with diethyl ether. The organic phases were combined, dried over sodium sulfate, filtered, and concentrated for GC–MS analysis [44].

**Synthesis of pyridylmethyl esters:** For esterification with 3-pyridinemethanol, freshly distilled oxalyl chloride (20 µL) was added to the gland extract (100 µL). After one day, excess oxalyl chloride and the solvent were evaporated under N_2_ and the sample was redissolved in DCM. For esterification, catalytic amounts of 4-(dimethylamino)pyridine (DMAP) and freshly distilled 3-pyridinemethanol (1 drop) were added. The reaction mixture was heated to 60 °C for 1 h and filtered. The filtrate was washed three times with H_2_O, the organic phases were dried over sodium sulfate and filtered before GC–MS analysis [8].

**Silver nitrate column chromatography:** Silver nitrate column chromatography was carried out as described [45]. An aq solution of silver nitrate (5.5 g) in H_2_O (30 mL) was added to silica gel (50 g). Water was added to barely cover the silica gel. The silica was stirred with a glass rod and shaken for 15 min and then placed in an oven at 100 °C until the silica gel was completely dried. The impregnated silica gel was stored in the dark until use.

### Syntheses

**1-(1-Propen-1-yl)piperidine (2):** In a manner analogous to [27], potassium carbonate (2 g, 15 mmol), piperidine (10 mL, 0.1 mol), and freshly distilled propanal (**1**, 2.1 mL, 30 mmol) were added to a 50 mL flask. The mixture was stirred at 0 °C for 1 h. The residue was filtered and washed twice with diethyl ether, and the solvent was removed in vacuo. The crude product was used without further purification. EIMS (70 eV) *m*/*z* (%): 125 (40), 124 (35), 110 (100), 96 (25), 82 (20), 68 (40), 55 (15), 41 (45).

**Methyl 4-methyl-5-oxopentanoate (3):** Compound **2** and methyl acrylate (6 mL, 66 mmol) were added to acetonitrile (20 mL). The mixture was heated to reflux for 20 h. A solution (10 mL) of acetic acid (5 g) and sodium acetate (5 g) in H_2_O (20 mL) were added and the reaction mixture was stirred for another 15 min. The phases were separated and the aq phase was extracted three times with diethyl ether. The combined organic phase was washed with brine, dried over sodium sulfate, and the solvent was removed, and the product was purified by column chromatography [28]. Yellow oil: 1.95 g (45% over 2 steps); ^1^H NMR (CDCl_3_, 300 MHz) δ 9.64 (s, 1H), 3.68 (s, 3H), 2.40 (m, 3H), 2.07 (m, 1H), 1.70 (m, 1H), 1.13 (d, *J* = 7 Hz, 3H); ^13^C NMR (CDCl_3,_ 75 MHz) δ 200.9, 173.4, 51.6, 45.5, 31.2, 25.3, 13.2; EIMS (70 eV) *m*/*z* (%): 116 (10), 113 (15), 112 (15), 85 (15), 74 (100), 59 (25), 55 (60), 43 (60)

**Heptyltriphenylphosphonium iodide:** In a manner similar to [46] triphenylphosphine (1.4 g, 5 mmol) was dissolved in acetonitrile (15 mL). Iodoheptane (1.3 mL, 8 mmol) was added, and the solution was heated to reflux for 1 h and then stirred for 18 h at room temperature. The solvent was removed and the residue was washed twice with pentane and dried under vacuum. The product was used without further purification. Analytical data were identical to those reported earlier.

**1-Methylheptyltriphenylphosphonium iodide (5):** In a manner similar to [31] heptyltriphenylphosphonium iodide (1.95 g, 4 mmol) was dissolved in dry THF (15 mL). A solution of *n-*butyllithium in hexane (1.6 M, 4.5 mL, 7 mmol) was added and the reaction mixture was stirred at 0 °C for 1 h. Methyl iodide (1.3 mL, 20 mmol) was added dropwise and the reaction mixture was stirred for a further 2 h. The solvent and excess methyl iodide were removed in vacuo. Analytical data were identical to those reported [31].

**Methyl 4,6-dimethyldodec-5-enoate** (**6**)**:** Phosphonium salt **5** (185 mg, 0.4 mmol) was dissolved in dry, degassed THF (10 mL). LiHMDS in hexane (1 M, 0.6 mL, 0.6 mmol) was added slowly at −10 °C and the resulting solution was stirred for 1 h at that temperature. Aldehyde **3** (105 mg, 0.7 mmol) was added at −10 °C and stirred for a further 18 h. The reaction mixture was diluted with pentane and filtered. After the removal of the solvent, the crude product was purified by column chromatography using pentane and diethyl ether (100:2) and obtained as a 1:1 mixture of diastereomers. This was followed by silver nitrate column chromatography using pentane and ethyl acetate (97.5:2.5) to enrich one diastereomer (de(*E*) = 50%). The NMR data were in agreement with published values [31]. Colorless oil: 15 mg (18%); ^1^H NMR (CDCl_3_, 500 MHz) δ 4.83 (d, *J =* 12 Hz, 1H), 3.65 (s, 3H), 2.40–2.30 (m, 1H), 2.3–2.2 (m, 2H), 2.05–1.90 (m, 2H), 1.66 (d, *J =* 13 Hz, 1.2H), 1.56 (d, *J =* 13 Hz, 1.8H), 1.38–1.21 (m, 10H), 0.93 (d, *J =* 7 Hz, 1.2H), 0.93 (d, *J =* 1.8 Hz, 1.8H), 0.88 (t, *J =* 14 Hz, 3H); ^13^C NMR (CDCl_3,_ 125 MHz) δ 174.5, 135.0, 129.8, 51.4, 39.7, 32.6, 31.9, 31.8, 31.8, 29.8, 28.9, 27.9, 23.4, 22.6(*Z*), 21.5, 21.3, 16.1(*E*), 14.1; EIMS (70 eV) *m*/*z* (%): 240 (15), 166 (10), 155 (60), 123 (40), 109 (35), 97 (40), 95 (75), 83 (60), 69 (100), 55 (75), 41 (35); IR (GC-IR) *Z*-isomer: v_max_ 2956, 2925, 2856, 1741, 1456, 1438, 1357, 1276, 1195, 1170, 1061, 986, 901, 851, 725 cm^−1^; *E*-isomer: v_max_ 2956, 2925, 2856, 1741, 1456, 1438, 1372, 1294, 1262, 1195, 1167, 1060, 989, 859, 729 cm^−1^.

## Supporting Information

File 1 Mass spectra, compound lists, synthetic procedures, NMR spectra.

## Data Availability

The mass spectra shown in the figures will be made publicly available after publication in the public data repository MACE. http://www.oc.tu-bs.de/schulz/html/MACE.html
