# Supplementary material for: The scent gland composition of the Mangshan pit viper, Protobothrops mangshanensis
Source: Beilstein J Org Chem. 2024 Oct 18;20:2644–54. doi: 10.3762/bjoc.20.222 (PMC11496706; doi:10.3762/bjoc.20.222)
Supplement: File 1 — Mass spectra, compound lists, synthetic procedures, NMR spectra. [file Beilstein_J_Org_Chem-20-2644-s001.pdf]

BEILSTEIN JOURNAL OF ORGANIC CHEMISTRY

Supporting Information

for

The scent gland composition of the Mangshan pit viper, *Protobothrops mangshanensis*

Jonas Holste, Paul Weldon, Donald Boyer and Stefan Schulz

Beilstein J. Org. Chem. **2024,** *20,* 2644–2654. doi:10.3762/bjoc.20.222

Mass spectra, compound lists, synthetic procedures, NMR spectra

License and Terms: This is a supporting information file under the terms of the Creative Commons Attribution License (https://creativecommons.org/licenses/by/4.0). Please note that the reuse, redistribution and reproduction in particular requires that the author(s) and source are credited and that individual graphics may be subject to special legal provisions.
The license is subject to the *Beilstein Journal of Organic Chemistry* terms and conditions: (https://www.beilstein-journals.org/bjoc/terms)

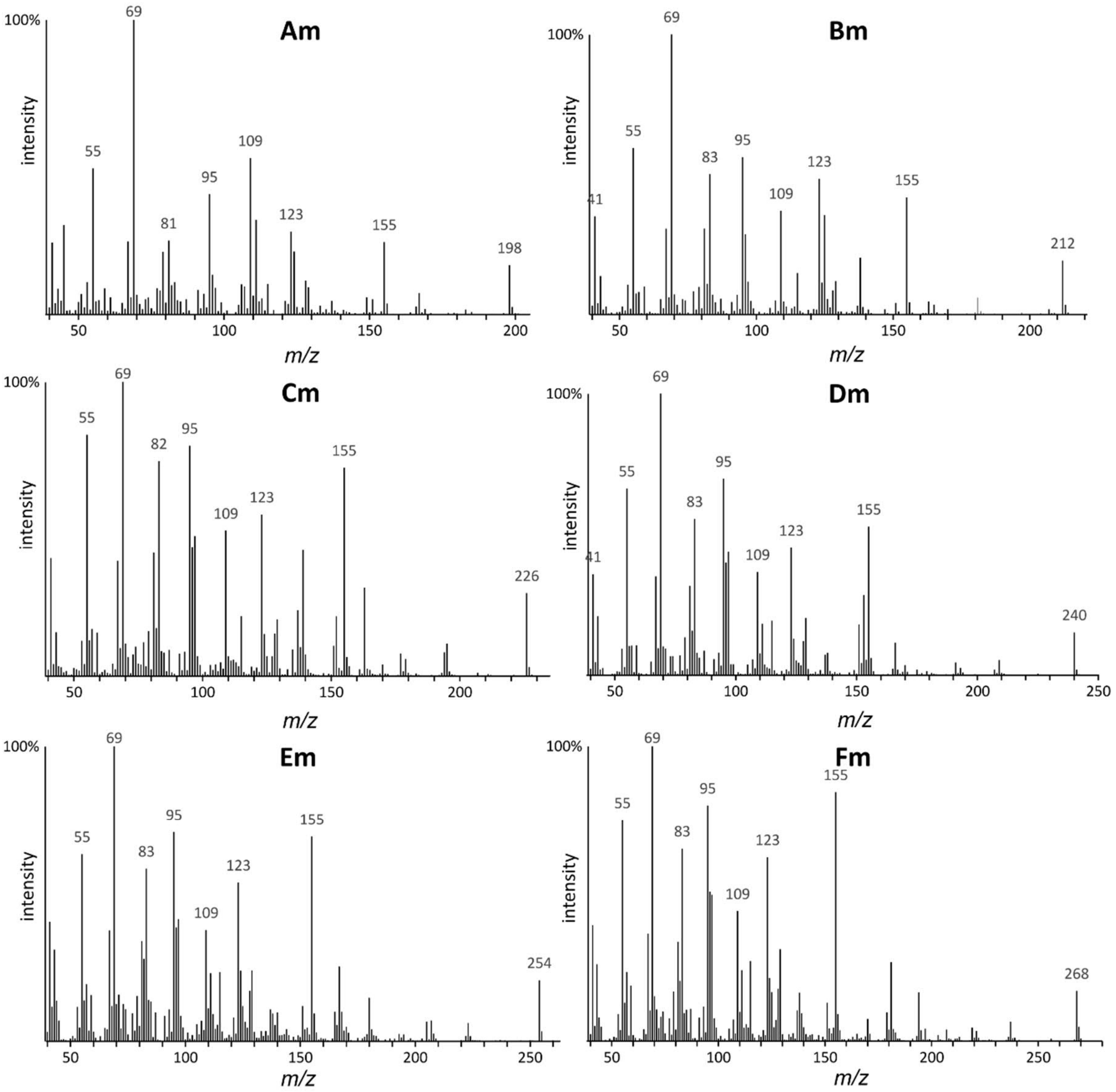

Figure S1. Mass spectra of methyl ester derivatives of unknown compounds **A-C.** Associated methyl esters are named **Am-Fm**. **Am**: methyl 4,6-dimethylnon-5-enoate, **Bm**: methyl 4,6-dimethyldec-5-enoate, **Cm**: methyl 4,6-dimethylundec-5-enoate, **Dm**: methyl 4,6-dimethyldodec-5-enoate, **Em**: methyl 4,6-dimethyltridec-5-enoate, **Fm**: methyl 4,6-dimethyltetradec-5-enoate.

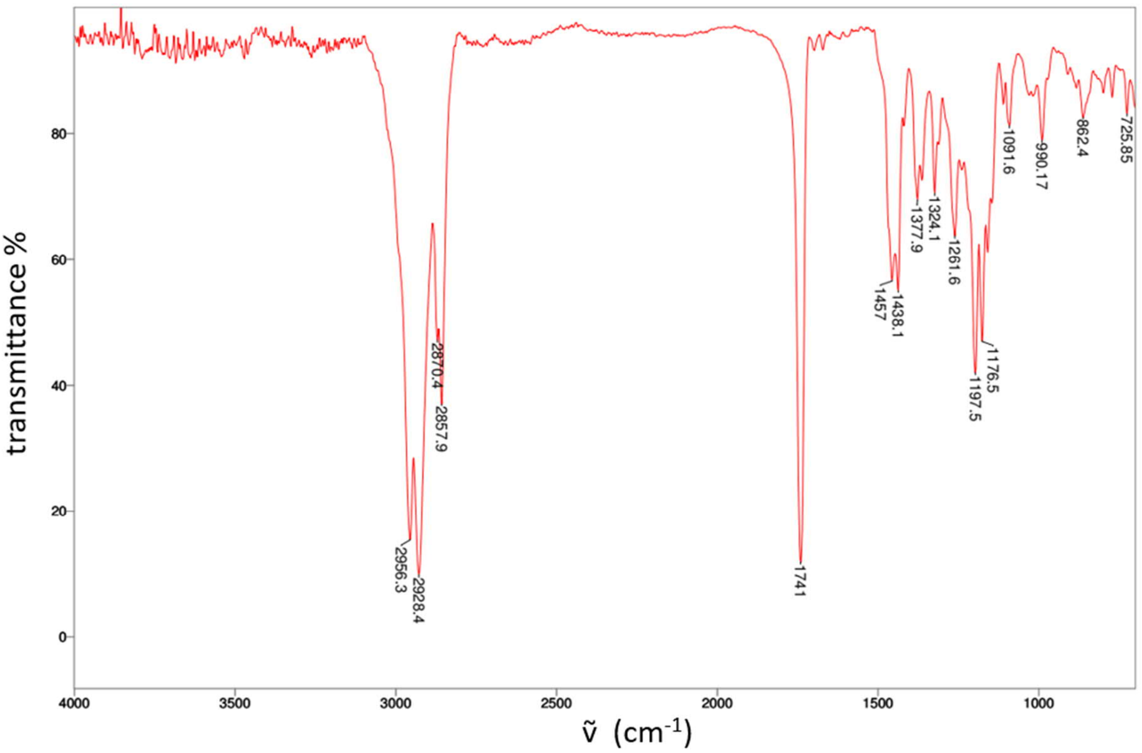

Figure S2. IR spectrum of **Dm**, methyl 4,6-dimethyldodec-5-enoate, the methylated natural compound **D**, obtained by GC/IR.

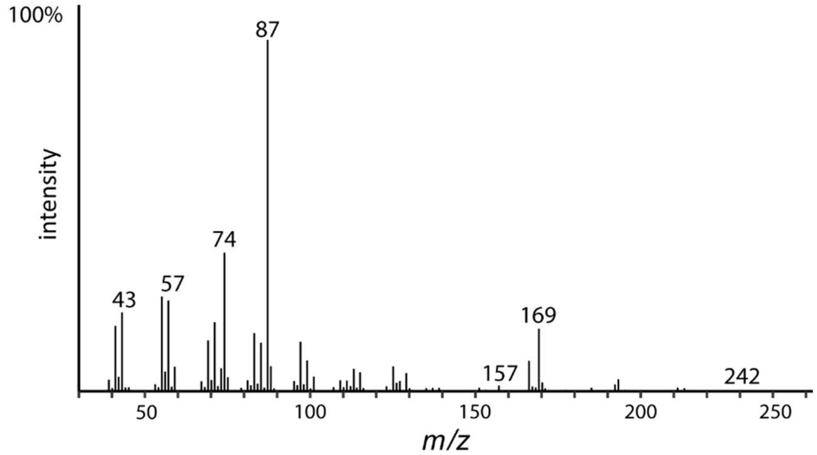

Figure S3. Mass spectrum of **Dmh**, methyl 4,6-dimethyldodecanoate, the methylated and hydrogenated natural compound **D**.

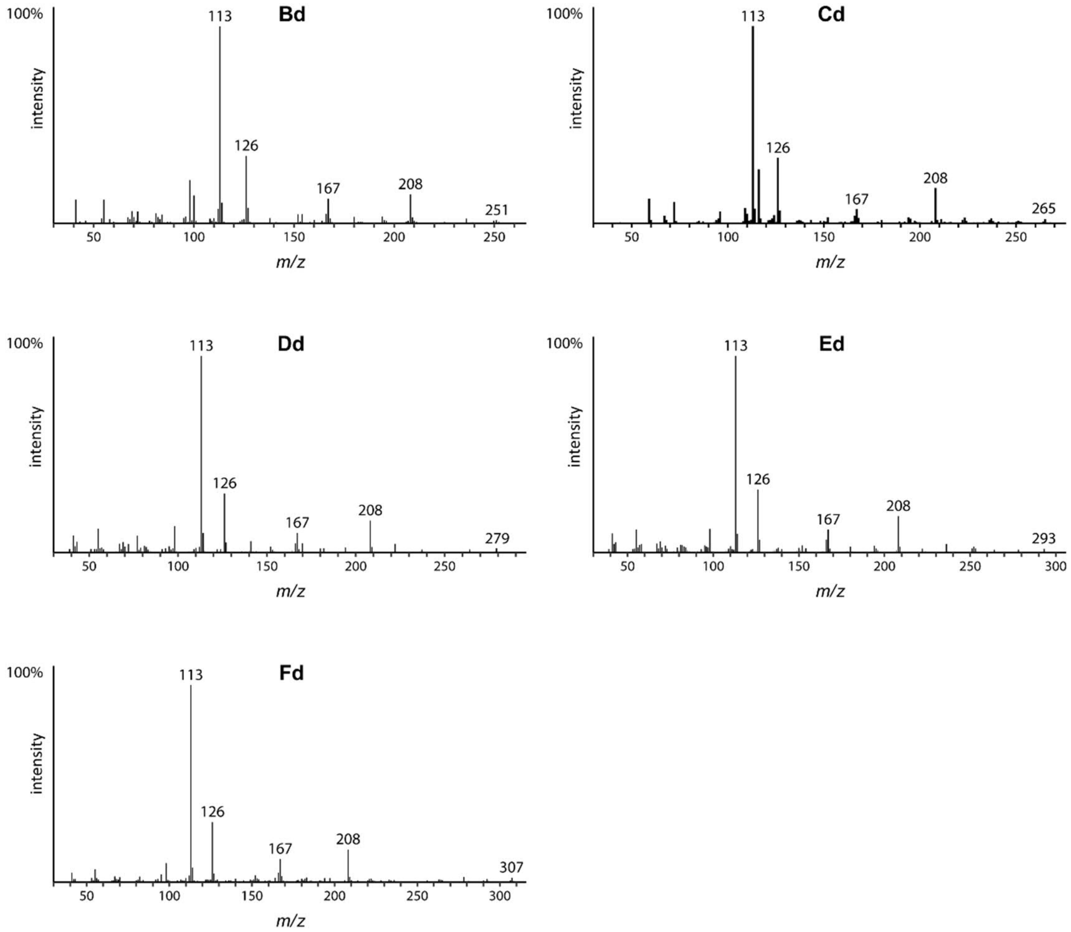

Figure S4. Mass spectra of DMOX derivatives of unknown compounds **B**-**F**. Associated dimethyloxazoline (DMOX) derivatives were obtained from derivatives of 4,6-dimethyldec-5-enoic (**Bd**), 4,6-dimethylundec-5-enoic (**Cd**), 4,6-dimethyldodec-5-enoic (**Dd**), 4,6-dimethyltridec-5-enoic (**Ed**), and 4,6-dimethyltetradec-5-enoic **(Fd)** acids.

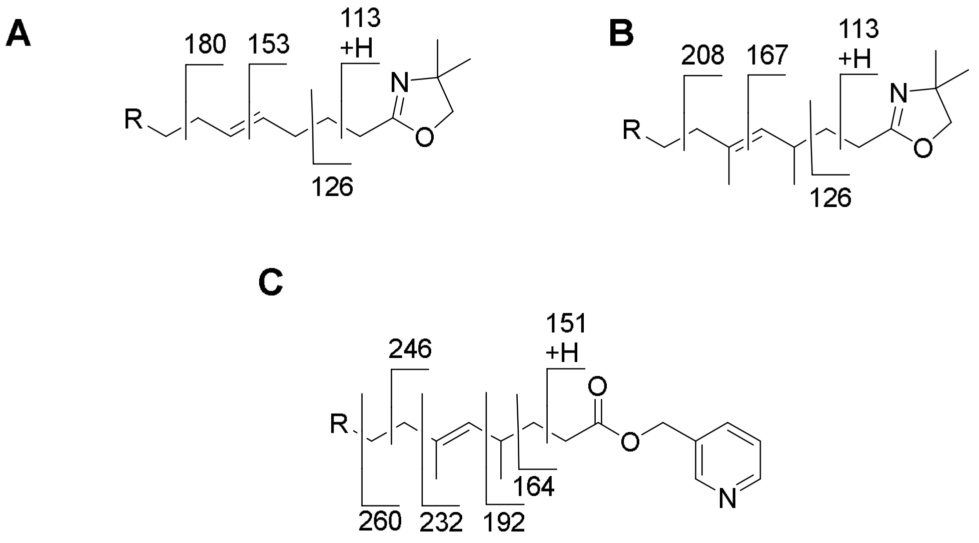

Figure S5. Mass spectrometric fragmentation of DMOX derivatives of 5-enoic acids (**A**), the fragmentation of the target compounds DMOX derivatives (**B**), and pyrididylmethyl esters (**C**). Please note that the formal assignment of ions used here does not imply the actual mechanism by which these ions are formed, e. g. direct cleavage of double bonds, which is unlikely.

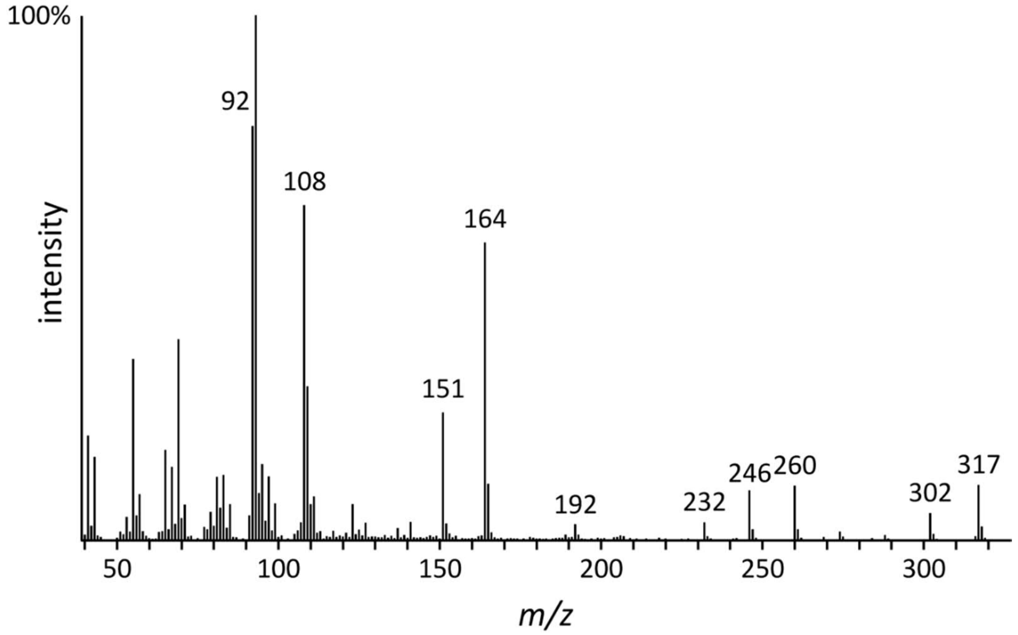

Figure S6. Mass spectrum of the pyridylmethyl ester of 4,6-dimethyldodec-5-enoic acid (**Dp**).

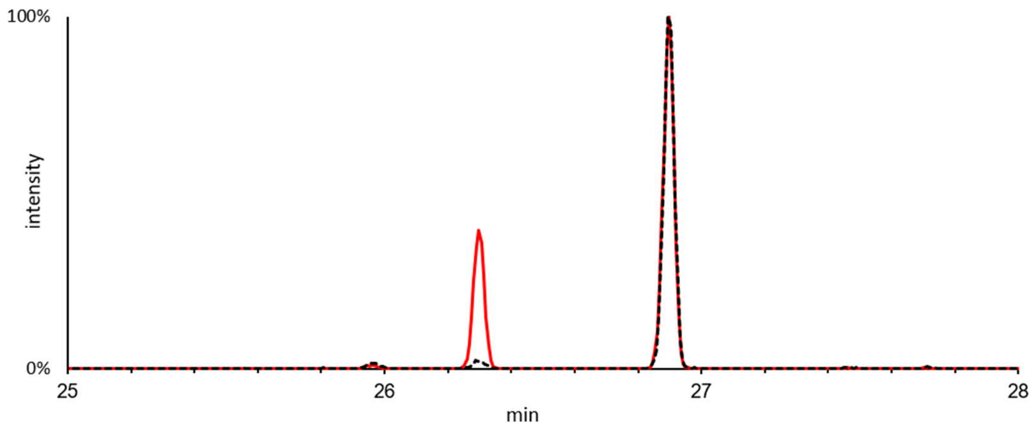

Figure S7. TIC chromatogram of the co-injection of a methylated natural extract with compounds **Dm** and **Dm'** (black) and synthetic methyl 4,6-dimethyldodec-5-enoate (**6** red).

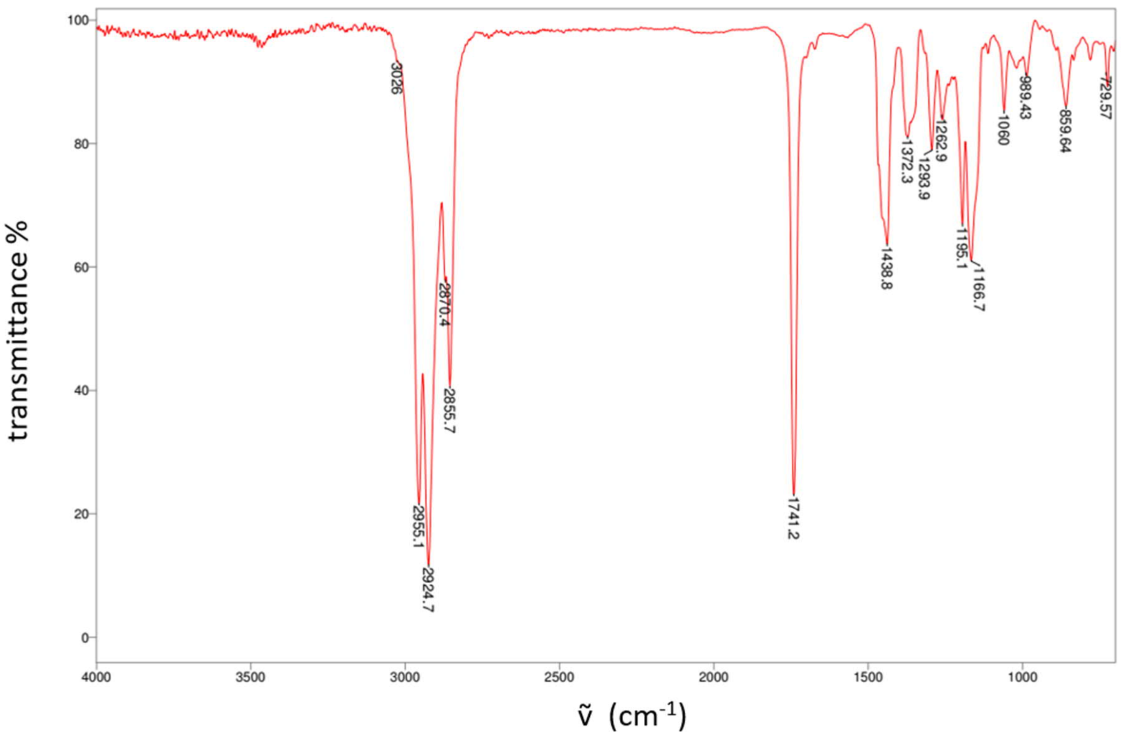

Figure S8. The IR spectrum of methyl (*E*)-4,6-dimethyldodec-5-enoate, obtained by GC/IR.

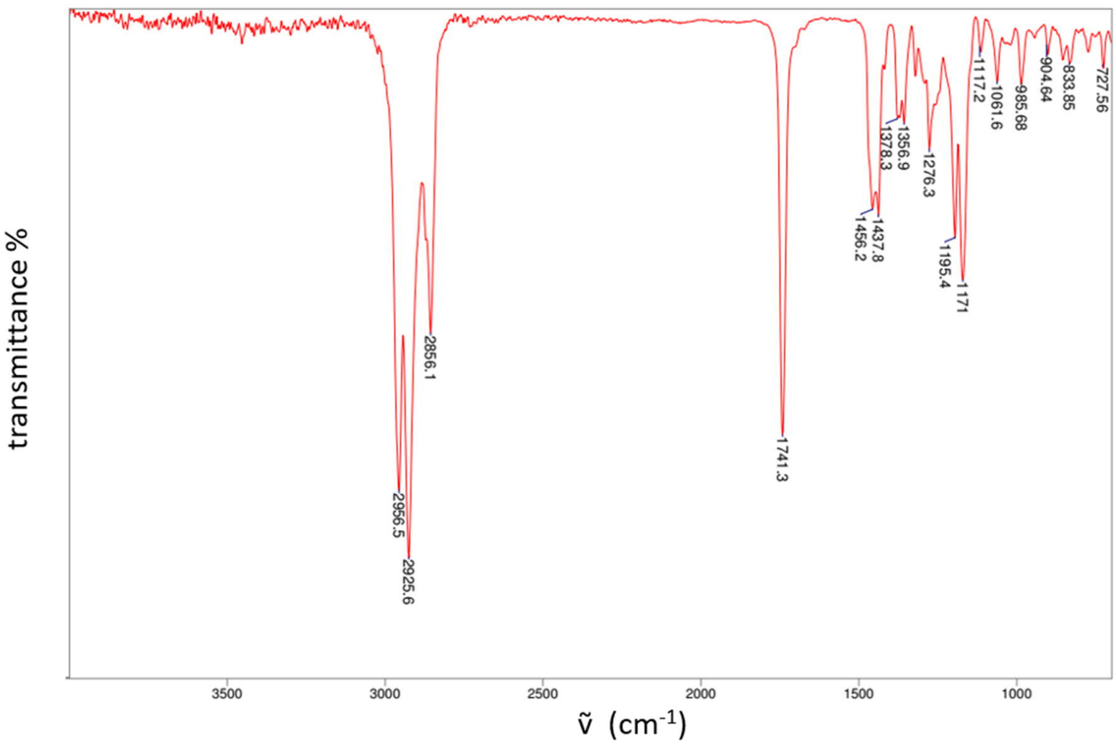

Figure S9. IR spectrum of methyl (*Z*)-4,6-dimethyldodec-5-enoate, obtained by GC/IR.

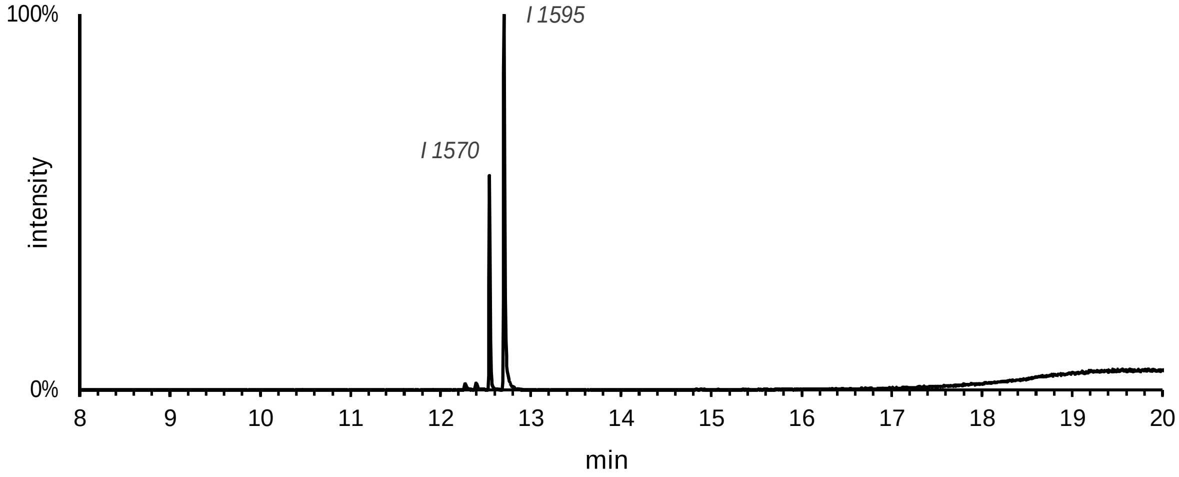

Figure S10. TIC chromatogram of enriched diastereomer mixture of **6** obtained by $AgNO_3$ column chromatography.

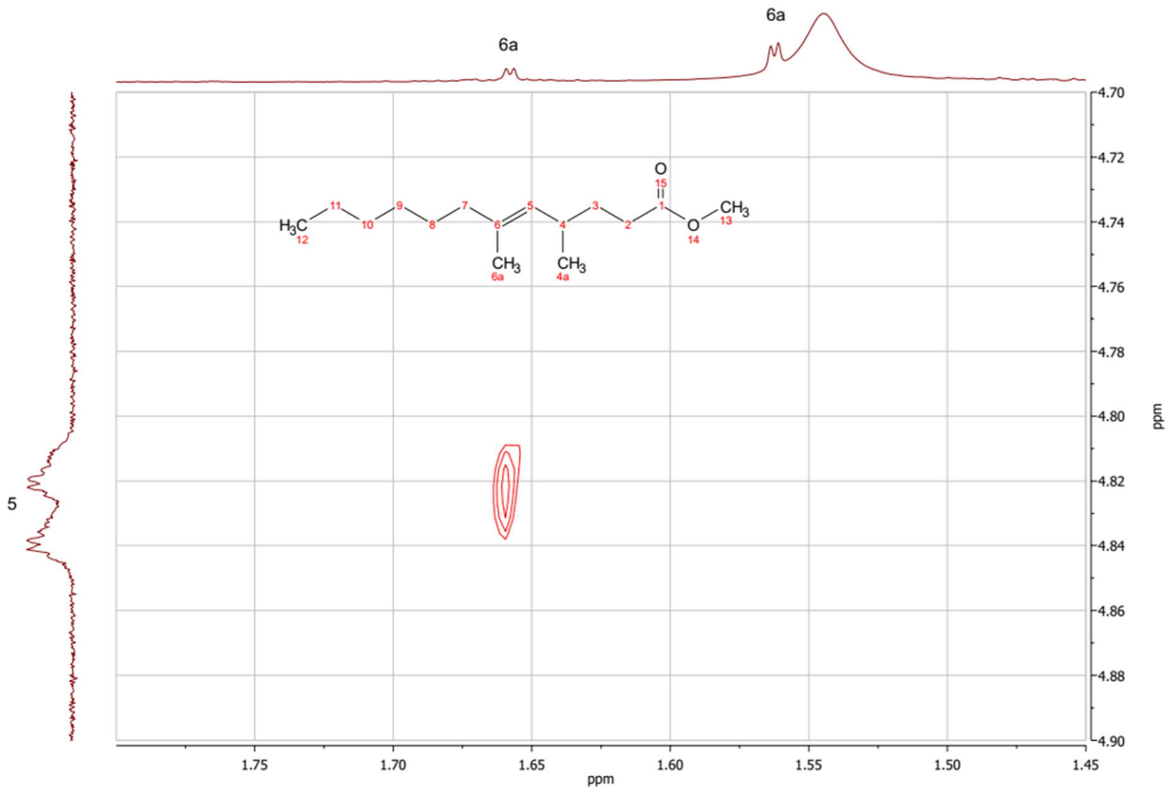

Figure S11. NOESY NMR spectrum of H-5 of methyl 4,6-dimethyldodec-5-enoate. The isomer *Z*-**6** shows coupling with 6a at 1.66 ppm while for *E*-**6** such a coupling with 6a at 1.56 ppm is absent, indicating the *trans*-configuration.

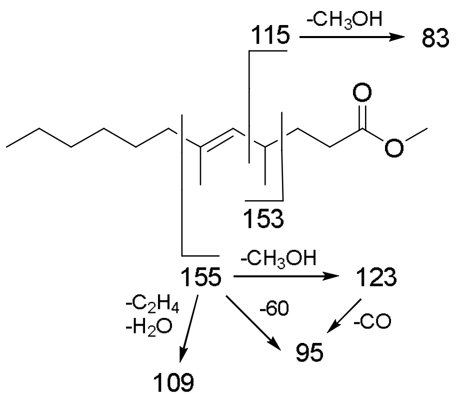

Figure S12. Proposed mass spectrometric fragmentation of methyl ester **6**.

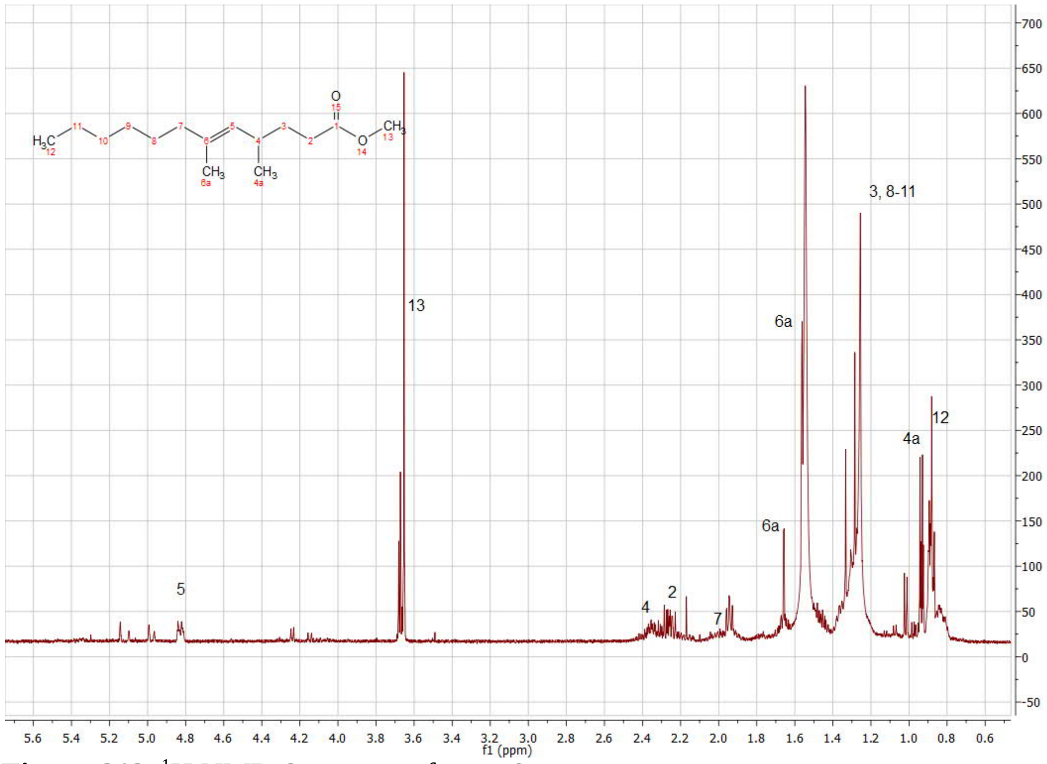

Figure S13. ^{1}H NMR-Spectrum of ester **6**.

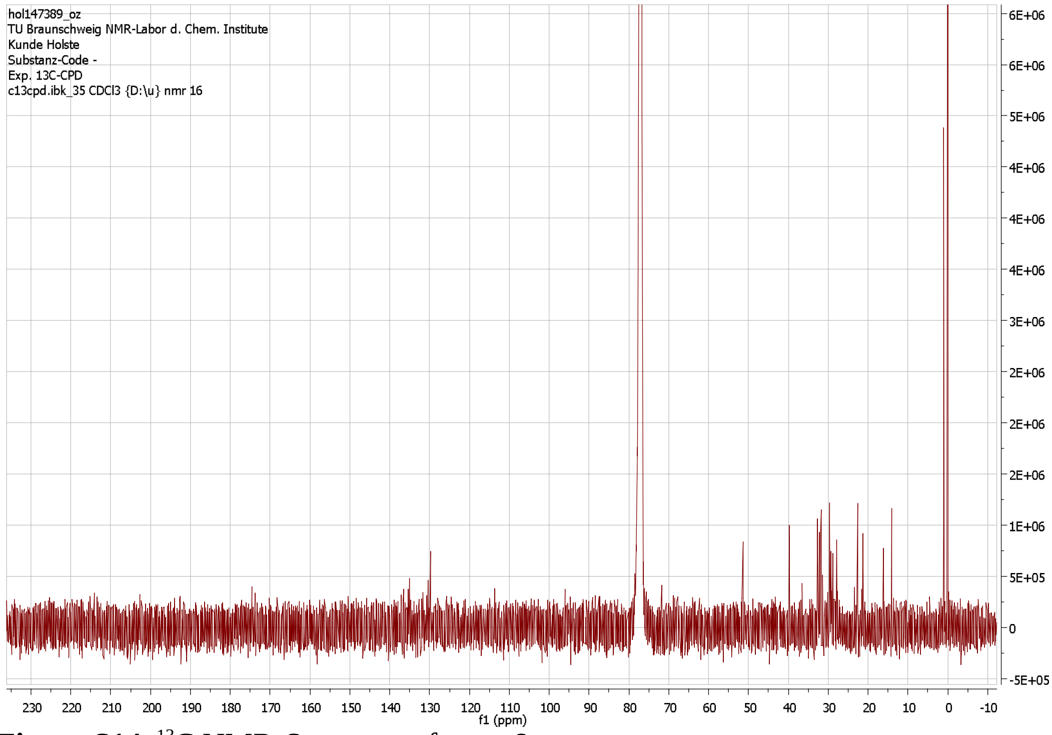

Figure S14. ^{13}C NMR-Spectrum of ester **6**.
